# Floral traits underlying mating system differentiation in the wind-pollinated sister species *Oryza rufipogon* and *Oryza nivara*

**DOI:** 10.1093/aobpla/plae073

**Published:** 2024-12-31

**Authors:** Michael Grillo, Andrés Gutiérrez

**Affiliations:** Department of Biology, Loyola University Chicago 1032 W. Sheridan Rd. Chicago, IL 60660, United States; Department of Biology, Loyola University Chicago 1032 W. Sheridan Rd. Chicago, IL 60660, United States

**Keywords:** Plant mating system, outcrossing, self-fertilization, autogamy, geitonogamy, wind pollination, *Oryza*

## Abstract

The shift from outcrossing to predominantly selfing is one of the most common transitions in plant evolution. This evolutionary shift has received considerable attention from biologists; however, this work has almost exclusively been focused on animal-pollinated systems. Despite the seminal ecological and economic importance of wind-pollinated species, the mechanisms controlling the degree of outcrossing in wind-pollinated taxa remain poorly understood. As a first step toward addressing this issue, we have conducted a comparative study of floral biology between two recently diverged sister species, *Oryza rufipogon* and *Oryza nivara* (Poaceae), that are wind-pollinated and possess distinct mating systems with *O. rufipogon* being outcrossing and *O. nivara* highly self-fertilized Therefore, these species present an ideal system for exploring mating system evolution in wind-pollinated taxa. We have identified key floral traits that differ between populations of these species and that are associated with mating system divergence including anther length, anther basal pore size, stigma papillae density, panicle shape, panicle exsertion, pollen viability, and early anther dehiscence. Of these traits, large anther basal pore size and early anther dehiscence are hypothesized to confer reliable autogamous selfing in *O. nivara*. Manipulations of floret number were conducted to partition the role of geitonogamy and autogamy in conferring self-fertilization. This experiment revealed that selfing in *O. nivara* is consistent with autogamous selfing, whereas *O. rufipogon* achieves selfing through geitonogamy. This study serves as a model for understanding the floral mechanisms controlling the outcrossing rate in other wind-pollinated systems, most notably other grasses.

## Introduction

Angiosperms display a remarkable diversity of reproductive traits and strategies. Understanding the evolutionary mechanisms that have shaped this diversity has been a central goal in studies of plant evolution. In particular, the mating system shift to predominant selfing from outcrossing is regarded as the most common transition in plant evolution and has been the subject of immense investigation (Barrett 2010a, Tsuchimatsu and Fujii 2022).

For nearly 150 years, the vast body of literature devoted to plant mating systems and pollination biology has focused almost exclusively on animal-pollinated systems (Friedman and Barrett 2009b, Barrett 2010b). This is unfortunate given that approximately 10% of all angiosperms are wind-pollinated (Ackerman 2000), including our most important crops (rice, wheat, maize, etc.), and wind pollination is a derived state, having evolved at least 65 times from animal-pollinated ancestors (Linder 1998, Rech *et al*. 2016). There are also intrinsic differences in floral biology between wind- and animal-pollinated species that are particularly interesting to study within a mating system context, including: (i) the presence/absence of attractive floral structures that evolve with the outcrossing rate, (ii) fundamental differences in floral anatomy that may determine the functional traits that control selfing, and (iii) the role of geitonogamy (mating between flowers of the same plant) and mechanisms that provide this mode of selfing.

In animal-pollinated systems, comparative studies of floral biology between selfing and outcrossing groups have been conducted in a wide range of species (Darwin 1876, Ritland and Ritland 1989, Lyons and Antonovics 1991, Goodwillie 1999, Armbruster *et al*. 2002, Vallejo- Marin and Barrett 2009, Huang *et al*. 2019, Frazee *et al*. 2021). Through these efforts, it has been well established that selfing plants produce small flowers with highly reduced attractive floral traits (e.g. showy petals, nectar production, attractive scents, etc.) when compared to outcrossers (Ornduff 1969, Tang and Huang 2007, Goodwillie *et al*. 2010, McElderry *et al*. 2022). To explain this syndrome, sex allocation theory has been developed, which predicts reduced allocation, to both male function and attractive structures, as selfing increases (Charlesworth and Charlesworth 1987, Charlesworth and Morgan 1991). Correlations between attractive floral characters and functional traits that provide selfing are common and can influence, or even limit, an evolutionary response to selection (Conner 2006). In wind-pollinated plants, attractive floral traits are largely absent; therefore, the transition to a selfing syndrome may be relatively less complex than in animal-pollinated species. Comparative studies of floral biology between selfing and outcrossing groups are lacking in wind-pollinated plants.

In addition to comparative work, more mechanistic experiments have been aimed at elucidating the mechanisms of self-fertilization in animal-pollinated taxa. In hermaphroditic self-compatible plants, the most common means for achieving autogamous (fertilization within an individual flower) selfing is through a reduction in herkogamy (Ritland and Ritland 1989, Dole 1990, Holtsford and Ellstrand 1992, Kohn and Barrett 1994, Belaoussoff and Shore 1995, Karron *et al*. 1997, Chang and Rausher 1999, Kalisz *et al*. 1999, Barrett 2003, Wessinger and Hileman 2016). Conversely, herkogamy in wind-pollinated groups has been unexplored, and its potential to contribute to selfing is unclear due to basic differences in floral biology, namely dry, unclumped pollen (Crane 1986, Ackerman 2000) and in the case of grasses, poricidal anthers that arrange pollen in a unique fashion and release it through terminal pores (Kirpes *et al*. 1996). Therefore, physical contact between stigma and anthers may not provide a selfing mechanism as reliable as that seen in animal-pollinated plants.

In wind-pollinated groups, inflorescence architecture is the trait that has received the most attention in regard to floral biology (Niklas 1985, 1987, Friedman and Harder 2004, 2005, Weller *et al*. 2006, Cresswell *et al*. 2010, Harder and Prusinkiewicz 2012). Poaceae taxa, in particular, display a diversity in the ‘openness’ of inflorescences ranging from diffuse to compact (Doust and Kellogg 2002, Koppolu and Schnurbusch 2019). Research involving inflorescence architecture in grasses has focused solely on wind aerodynamics and strategies for pollen removal and receipt and has not been investigated in terms of selfing rates. It is likely that the close proximity of flowers in compact inflorescences could enhance the opportunity for geitonogamy.

Self-fertilization can arise from either geitonogamy or autogamy. Large floral displays function in attracting pollinators across long distances but can result in geitonogamy when pollinators visit subsequent flowers on the same plant. Geitonogamy can be quite common in animal-pollinated systems (Harder and Barrett 1995, Eckert 2000, Dorken *et al*. 2002, Karron *et al*. 2004) and is suspected to promote the evolution of dichogamy or dioecy, particularly in large plants with abundant flowers (Barrett 2003). Similarly, wind could provide geitonogamous selfing between flowers in close proximity. Friedman and Barrett (2009a) have identified substantial geitonogamous selfing in *Carex* spp., and propose that, unlike animal-pollinated species, geitonogamy in wind-pollinated systems may often provide reproductive assurance, particularly in plants with unisexual flowers. More experiments in wind-pollinated plants are needed that dissect the contributions of autogamy and geitonogamy, in order to understand their role in selfing.

Here, we present a comparative study of floral biology between two wind-pollinated sister species, *Oryza nivara* and *Oryza rufipogon*, that have recently diverged in their mating system (Morishima and Barbier 1990, Vaughan 1994, Xu *et al*. 2020). The sister species are wetland grasses distributed throughout the tropics and subtropics of Asia. These species are sympatric throughout the range of *O. nivara* but utilize distinct habitats (Zheng and Ge 2010). *Oryza nivara* is an annual that inhabits ephemeral pools containing standing water throughout the monsoon season but then desiccates during the dry season (Vaughan 1994, Xu *et al*. 2021). Conversely, *O. rufipogon* is a perennial that inhabits lowland marshes and wetlands that retain sufficient water levels for the plants to persist throughout the year. Both species are self-compatible and hermaphroditic but differ in their mating system. Barbier (1989) measured multilocus outcrossing rates and found that *O. rufipogon* had substantially higher outcrossing (t_m_ = 0.50 and 0.56) than *O. nivara* (t_m_ = 0.04), which is consistent with earlier estimates based on other approaches (Oka and Morishima 1967). In the *Oryza* genus, the majority of species are perennial, which is the presumed ancestral trait with annual life history being derived independently multiple times (Grillo *et al*. 2009). Phylogenetic analysis has indicated that *O. nivara* has recently diverged from a common ancestor resembling *O. rufipogon* within the last 0.16 million years (Barbier *et al*. 1991, Zhu and Ge 2005), which is consistent with broader observations of annual selfers evolving from perennial outcrossers (Barrett *et al*. 1996). Thus, *O. nivara* and *O. rufipogon* present a novel opportunity for studying classic evolutionary transitions in plant mating systems within a wind-pollinated group. We hypothesize that *O. nivara* will possess floral traits that provide reliable self-fertilization, whereas *O. rufipogon* has trait values that promote outcrossing. The specific goals of this study are to characterize differences in floral biology that may contribute to the mating system and to partition the role of geitonogamy and autogamy in providing selfing. Additionally, we provide a mechanistic framework for the evolution of mating system divergence in these species. This work sets the stage for future studies aimed at understanding mating system evolution in wind-pollinated taxa.

## Methods

**Figure 1.**
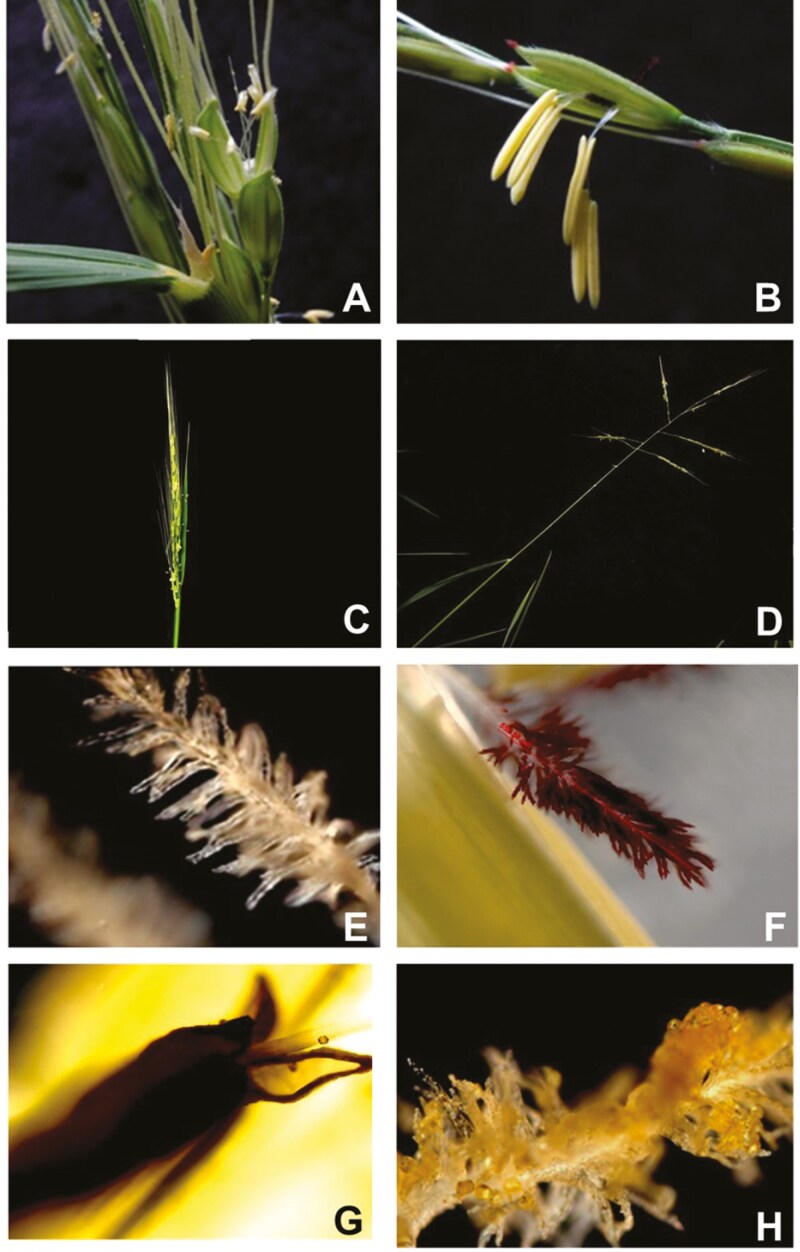
Functional traits controlling mating system differentiation in *Oryza nivara* and *Oryza rufipogon*; *O. nivara* traits are in the left column and *O. rufipogon* on the right; (A) *O. nivara* floret demonstrating anther size; (B) *O. rufipogon* floret demonstrating anther size; (C) *O. nivara* panicle shape and exertion; (D) *O. rufipogon* panicle shape and exertion; E) *O. nivara* stigma length, papillae density, and stigma colour; (F) *O. rufipogon* stigma length, papillae density, and stigma colour; (G) Basal pore of *O. nivara*; (H) early dehiscence of *O. nivara*, stigma coated in pollen prior to floret opening.

A comprehensive study of potential floral traits that may influence mating system divergence between *O. rufipogon* and *O. nivara* was conducted. Floral traits were identified through an extensive literature review in relevant systems and personal observations of each species in the greenhouse and in natural habitats. Floral traits that encompass both morphological and physiological traits including panicle openness, panicle exsertion, anther length, anther basal and apical pore size, filament length, pollen size, stigma length, stigma papillae density, stigma pigmentation, stigma receptivity, pollen viability, and dichogamy were included (Fig. 1). Here we present data for traits that were found to differ significantly between the focal species. The traits were examined in three typical accessions of each species that were provided by the International Rice Research Institute germplasm collection: *O. nivara* IRGC105771 (Thailand), IRGC104735 (Thailand), and IRGC80470 (India), and *O. rufipogon* IRGC80569 (India), IRGC80506 (India), IRGC105422 (Sri Lanka). For this study, a single genotype of each accession was cloned by sampling dividing tillers to generate numerous replicate individuals for trait measurements. All the putative mating system traits require plants to be in flower, and this was achieved by exposing plants to short-day lengths in the growth chamber.

### Floral morphological traits

Anther length, anther basal pore size, and stigma papillae number were measured from freshly collected samples with a compound light microscope. Length measurements were obtained with a lens ocular micrometre and were converted to the appropriate units (µm or mm). Stigma colour was measured qualitatively as purple or white. Panicle openness was measured by the angles between primary branches and rachis. Panicle exsertion was measured by the distance from the flag leaf node to the first primary branch node of the panicle. All traits included at least 10 measurements from different florets or panicles.

### Physiological traits

The timing of anther dehiscence was examined prior to anthesis. Under the constant conditions of the growth chamber, both species began flowering approximately 4 hours after first light. All florets of a panicle typically complete flowering within a 4-day period, thus each day a subset of florets open (Grillo per. obs.). On the final day of this period, approximately five florets remain and there is a very high probability that they will open on that day. Thus, it is reasonably possible to predict when a floret will open on the last day of the flowering period. When this was the case, we waited until the first floret opened for that day and then promptly opened the remaining florets manually with forceps. Anthers and stigmas of these florets were collected and examined under a light microscope to determine if anthers had dehisced and if there was pollen present on the stigma. Early dehiscence was scored as 0, 1, 2, 3, or 4 based on the relative number of pollen grains on the stigma prior to floret opening: (0 = 0, 1 = < 20, 2 = 20-40, 3 = 40-80, 4 = > 80 pollen grains, respectively).

Pollen viability was measured by pollen staining with a fluorescent dye following Rodriguez-Riano and Dafni (2000). Pollen was collected at the following time intervals (minutes following anthesis), 0, 15, 30, 45, and 60. To store pollen, anthers were harvested with forceps and then shaken over a microcentrifuge tube containing 1 ml of 10% sucrose. Storage of pollen in sucrose solution prevents further degradation and maintains pollen grains at the collection state (Rodriguez-Riano and Dafni 2000). A Fluorescein Diacetate (FDA) solution was created with 2 mg/ml of FDA in acetone. Droplets of FDA solution were added to the pollen collection tubes (suspended in 1 ml 10% sucrose) until the solution turned milky grey. The stained pollen was then allowed to incubate for 5 minutes, before being viewed on a fluorescent microscope (purple-indigo beam). Pollen grains were scored as being viable if they absorbed the stain. For each measurement, approximately 100 pollen grains were scored. Measurements at each time point were repeated at least five times by collecting pollen from fresh florets. Pollen staining indicates the presence of a cytoplasm and is merely a proxy for pollen viability (Rodriguez-Riano and Dafni 2000). To validate the pollen staining results, a pollination study was conducted. For this, approximately 30-minute old pollen was deposited on fresh stigmas (at anthesis) of one *O. rufipogon* accession (IRGC80506). The donor *O. rufipogon* florets were emasculated at anthesis prior to anther dehiscence to prevent self-pollination. Accomplishing this in *O. nivara* is highly unlikely and so this experiment was attempted only with *O. rufipogon* as the maternal plant. This experiment was conducted 10 times for each species.

### Autogamy vs geitonogamy

In order to distinguish between geitonogamy and autogamy, manipulations of floral number were conducted (Schoen and Lloyd 1992). To determine the rate of autogamy, panicles were trimmed with scissors so that only one floret remained. These plants were then isolated in separate greenhouse rooms and allowed to flower under which conditions only autogamous selfing is possible. Plants were marked on the day of anthesis, and approximately 1 week later, florets were collected to determine if there was successful self-fertilization by identification of a developing seed. To determine the rate of geitonogamy, plants were cut back so that only a single panicle was allowed to flower, and again plants were isolated from each other in separate greenhouse rooms. After all flowers had opened, the panicle was bagged, and the seed set was scored once the seeds had matured. Under these conditions (a single panicle with multiple flowers), there is the opportunity for both autogamy and geitonogamy. Additionally, plants with several panicles were allowed to flower in a greenhouse room with dozens of simultaneously flowering plants and abundant wind generated by fans, thereby allowing increased opportunity for geitonogamy. This floral manipulation study was conducted in one accession of *O. nivara* (IRGC80470) and *O. rufipogon* (IRGC80506) with at least five replicates for single and multi-panicle treatments and 10 florets in the single-floret treatment.

### Statistical analysis

All trait data were analysed using R Statistical Software (v 4.3.1; R Core Team 2023), and the package dplyr (v 1.1.2; Wickham *et al.* 2023) was employed to obtain central tendency measurements. Before utilizing parametric methods for data comparison, assumptions of normality were assessed using the Shapiro-Wilk test on the residuals following Analysis of Variance (ANOVA) in R. Additionally, homogeneity of variance among the compared data was evaluated using a Levene test implemented with the car package (v 3.1.2; Fox and Weisberg 2019). For the traits that met the normality and variance homogeneity requirements, an ANOVA analysis was conducted followed by Tukey’s multiple comparison test of means. For the dataset that did not meet the assumption requirements for parametric analysis, the Kruskal-Wallis rank sum test was performed, followed by Dunn’s multiple comparisons, and the *P*-values were adjusted using the Holm method. These analyses were conducted using the FSA package (v 0.9.5; Ogle *et al.* 2023 ). The results of multiple paired comparisons were visualized using the multcompView package (v 0.10.0; Graves *et al.* 2024). The results of the ANOVA and Kruskal-Wallis tests were summarized using the emmeans package (v 1.10.0; Lenth 2024). These results were utilized for constructing graphics with the ggpubr package (v 0.6.0; Kassambara 2022) and ggplot2 package (v 3.4.2; Wickham 2016). A multivariate analysis of variance (MANOVA) was conducted on paired traits that were collected simultaneously, and the data were traceable to the same panicle, flower, or flower component. Specifically, the traits anther length and basal pore, panicle exsertion and panicle angle, and papillae density and stigma length were paired to perform the MANOVA, with accession and species as independent variables. Before the analysis, the dependent variables were tested for normality using the Shapiro-Wilk test, performed via the mvnormtest package, v. 0.1-9-3 (Jarek 2024) in R. If the data did not meet the normality assumptions, a Box-Cox transformation was applied using the MASS package v. 7.3-60 (Venables and Ripley 2002). After transformation, for the traits that initially did not meet the normality requirements, a MANOVA test was conducted using base R. All code for statistical analysis is available on the Grillo lab github: https://github.com/grillolab/Oryza-mating-system-study.

## Results

**Figure 2.**
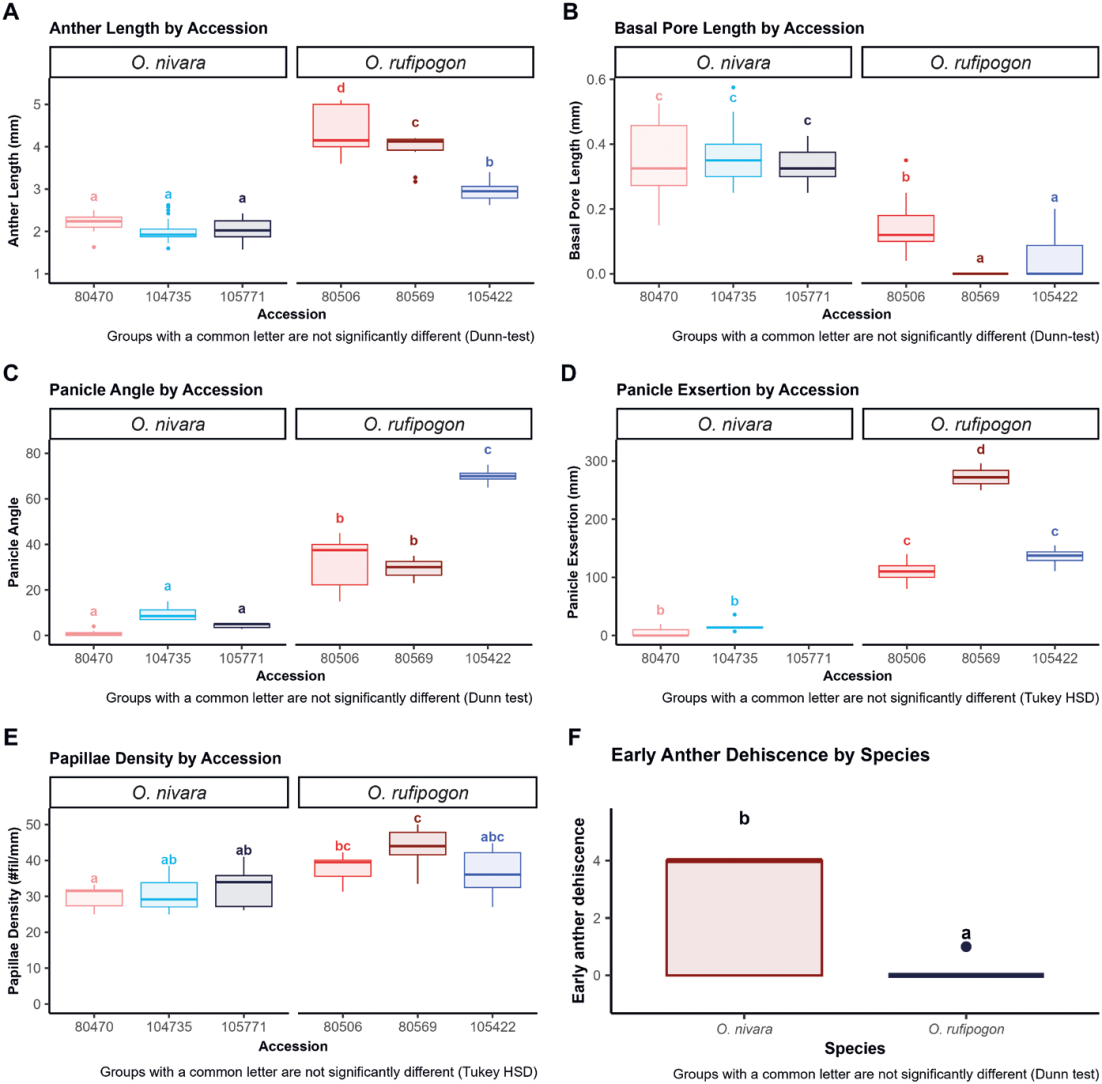
Floral trait data for *Oryza rufipogon* and *Oryza nivara* accessions: anther length (A), anther basal pore size (B), panicle angle (C), panicle exsertion (D), stigma papillae density, and early anther dehiscense (F). Means that are followed by a common letter are not significantly different according to the Tukey Honestly Significant Difference (HSD) or Dunn test. Early anther dehiscence (F) was measured in one accession of each species.

Through our observational analysis, the following traits displayed significant differences in phenotypic means between species as revealed through ANOVA: anther length, anther basal pore size, stigma papillae density, stigma pigmentation, pollen viability, early dehiscence, panicle openness, and panicle exsertion (Figs 1 and 2; Supplemental Fig. 1). Trait data are provided in Supplemental Table 1. Florets of *O. nivara* accessions had smaller anthers with a larger basal pore size and less stigma papillae in comparison to *O. rufipogon* (Fig. 1A, B, and E, respectively). In regard to panicles, *O. rufipogon* has more open panicles that are exserted further from the leaf (Fig. 1C and D, respectively). Due to data acquisition limitations, three separate MANOVAs were performed. The collection of certain traits impeded the measurement of others, and some measurements encompassed multiple other traits, making it difficult to establish a direct association between a single measurement of each trait. For instance, the measurement of panicle angle encompasses several other measurements, such as stigma density, anther length, and pollen diameter, among others. In all the MANOVA analyses performed, the independent variables were species and the specific accessions used in the analysis. The first group of dependent variables analysed were anther length and basal pore diameter. Consistent with the previous individual ANOVA analysis, the MANOVA results (Supplemental Table 2) indicated that species and accession were statistically significant in influencing the multivariate means of anther length and basal pore diameter. In other words, species and accession significantly affect the combination of these dependent variables. Similar results were obtained for the dependent variables pairs panicle exsertion and panicle angle, as well as for papillae density and stigma length.

Unlike *O. rufipogon*, early dehiscence occurred prior to floret opening in *O. nivara*, resulting in pollen deposited on the stigma (Fig. 2). Early dehiscence was scored based on the relative number of pollen grains deposited on the stigma prior to anthesis. *Oryza nivara* had an early dehiscence value of 2.33, compared to 0.1 in *O. rufipogon* (Fig. 1F). While this difference is significant, it is likely to be an underestimate for *O. nivara*. A measurement of ‘0’ could either indicate that the anthers did not dehisce and deposit pollen on the stigma, or that the particular floret was sampled too early to witness early dehiscence. In *O. rufipogon*, early dehiscence was observed in only two florets, and in both cases, few pollen grains were deposited. These instances could have been caused by contamination from pollen in the air, or physical damage of the stigma during manual flower opening. Nevertheless, even if this is not the case, early dehiscence occurs rarely in *O. rufipogon*.

In a time course study, the decay in pollen viability was much more pronounced in *O. nivara* accessions starting at 45 minutes (Supplemental Fig. 1). The ability of pollen grains to absorb a stain is merely a proxy for the actual pollen viability and is likely to be an overestimate (Song *et al*. 2004). A functional experiment was conducted by placing 30-minute-old pollen on fresh stigmas. In this experiment, there was no significant difference between species (mean of 60% and 70% in *O. nivara* and *O. rufipogon*, respectively). The large number of pollen grains deposited on the stigma may have allowed for seed production, despite the presumably low pollen viability of *O. nivara*.

**Figure 3.**
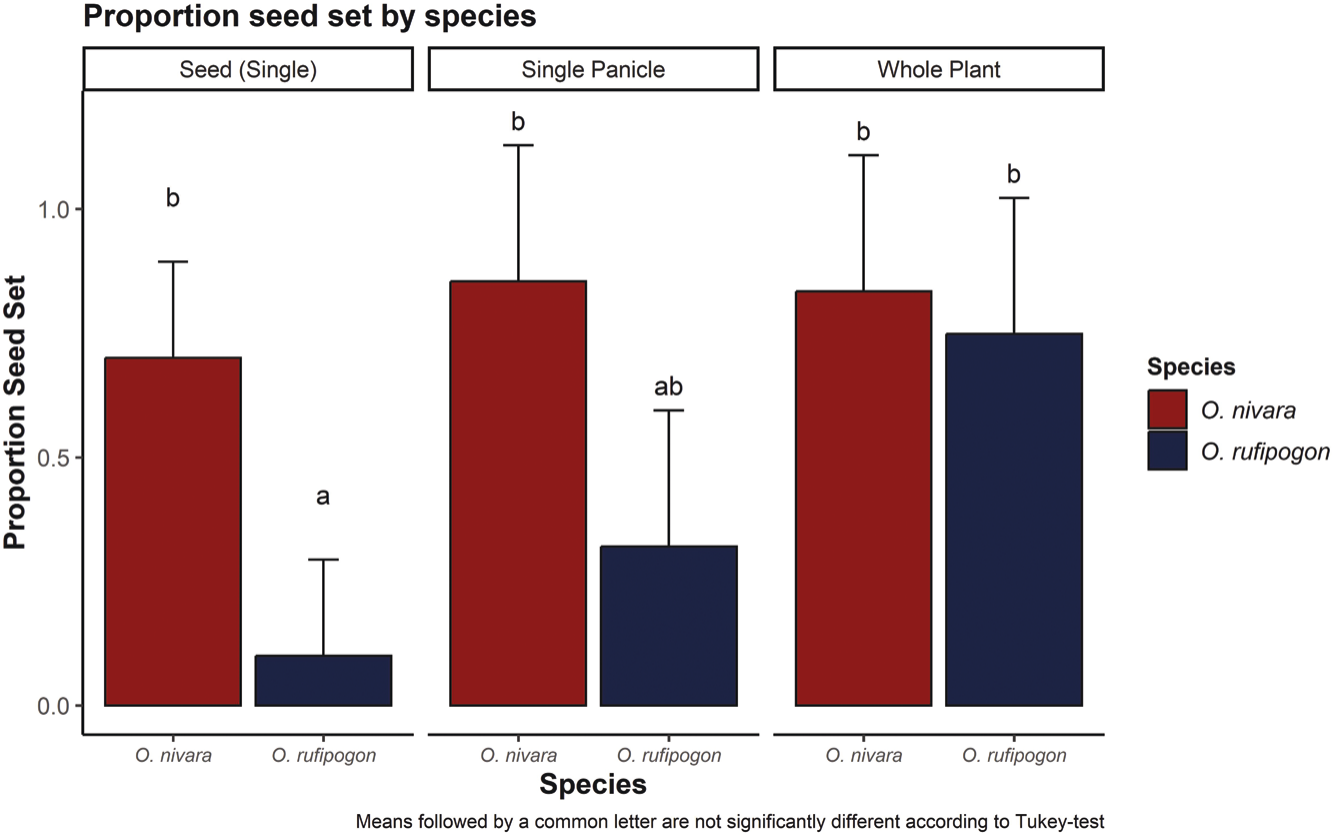
Partitioning of selfing into autogamy and geitonogamy through floral number manipulations; in the ‘single’ treatment, panicles were trimmed to a single floret, allowing only for autogamous selfing; in the ‘single panicle’ treatment, there is an opportunity for autogamy and geitonogamy; in the ‘whole plant’ treatment, numerous plants flowered simultaneously and so there was less pollen limitation with an opportunity for autogamy and geitonogamy.

By manipulating floret number, we can partition the selfing rate into contributions from geitonogamy and autogamy (Fig. 3). If autogamous selfing is predominant, one would predict a consistent level of seed set regardless of floret number. Alternatively, if geitonogamy is predominant, the seed set should increase with increases in floret number/pollen availability (Schoen and Lloyd 1992). In the treatments with only a single floret, the seed set averaged 70.0% and 10.0% in *O. nivara* and *O. rufipogon*, respectively. Since there is only one floret, all resulting seed set is generated through autonomous selfing. In the treatments with a single panicle and whole plants, there is the opportunity for geitonogamy and autogamy. Under these conditions, there is a significant increase in the average seed set for *O. rufipogon*, but not for *O. nivara*. These results clearly demonstrate that autogamy is the predominant form of selfing in *O. nivara.* While geitonogamy did provide additional seed set, this may be an artefact of damage induced by the floral manipulations. In the single-floret experiments, the panicle was cut back, which could have had detrimental effects on the remaining floret. Conversely, there were no such manipulations in the single-panicle experiment. This does not impact the interpretation of the role of autogamy between species, as both experienced the same manipulations in the single-floret experiments, but yielded drastically different results. In the case of *O. rufipogon*, geitonogamy increased seed set in the single-panicle experiment, although the effects were limited likely due to pollen limitation with the low number of florets open on each day. This is demonstrated in the whole plant treatment, where there was vastly more available pollen (Fig. 3). It should be noted that there is an opportunity for autogamy, geitonogamy, and outcrossing (with different plants of the same genotype) in this treatment; however, this is inconsequential as pollen limitation is the key factor being addressed. Under these conditions, the seed set in *O. rufipogon* did not differ significantly from *O. nivara.*

## Discussion

One of the most common transitions observed in plant evolution is the shift from outcrossing to selfing mating systems. While understanding the mechanistic basis of this classic phenomenon in plant evolution has been a subject of intense scientific interest, very little attention has been devoted to wind-pollinated plants. To address this knowledge gap, we have conducted a comparative study of floral biology in two recently diverged sister species that differ in the degree of outcrossing. Overall, we find a suite of floral traits that are associated with the shift in mating system and are prime targets for future functional validation and exploration in other wind-pollinated systems. Additionally, we differentiate between the mode of self-fertilization by portioning the role of autogamy and geitonogamy. Below, we discuss these key results and provide a context for future experimentation.

Mating system traits

**Figure 4.**
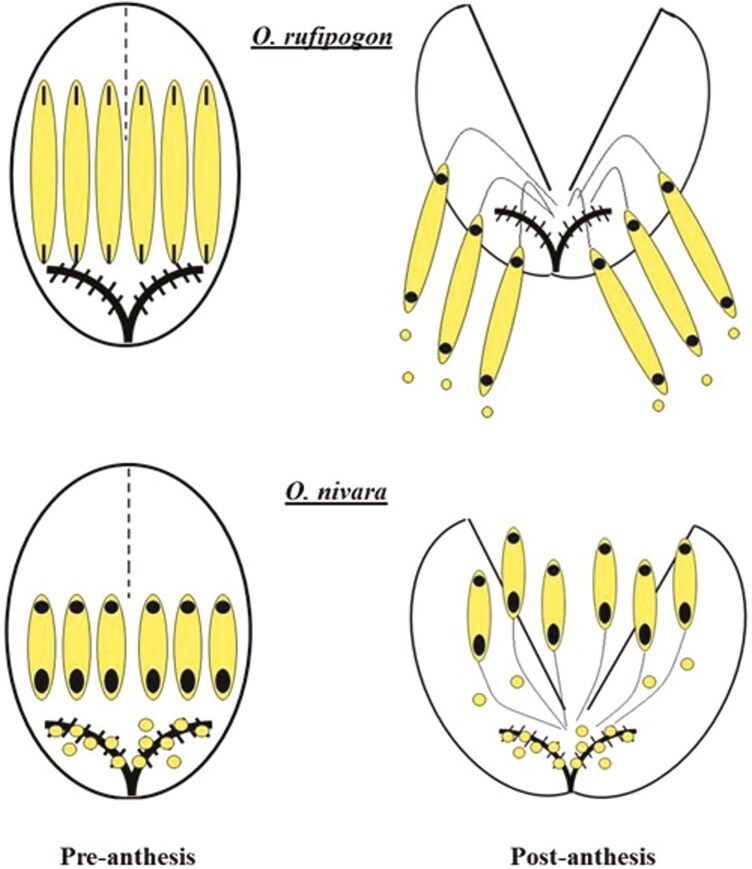
Floral models of *Oryza rufipogon* and *Oryza nivara*, pre and postanthesis; *O. nivara* has smaller anthers than *O. rufipogon* and the anther pores open, releasing pollen through the basal pore prior to anthesis; upon anthesis, the anthers remain upright and dispense pollen through the basal pore onto the stigma; *O. rufipogon* has much larger anthers that do not dehisce prior to anthesis; upon anthesis, the large basal pores hang pendent-like below the flower, and pollen is dispensed through the apical pore.

In wind-pollinated plants, male fitness is thought to be directly related to investment in pollen production (Charlesworth and Charlesworth 1981, Paterno *et al*. 2020), and because of this, male-biased sex allocation is commonly observed (Burd and Allen 1988). As an illustration, anthers of *O. rufipogon* are approximately 60% of the total flower length. Anthers of *O. nivara* are half the length of *O. rufipogon* (Fig. 2) and likely house half the amount of pollen. This decrease in anther length fits predictions of sex allocation theory, with decreased investment in male function as selfing increases (Charlesworth and Morgan 1991, Paterno *et al*. 2020). *Oryza rufipogon* and *O. nivara* possess poricidal anthers that open at pores on each end. There is no difference in apical pore size between these species. However, there is a considerable difference in basal pore size, with *O. nivara* having a basal pore twice that of *O. rufipogon* (Fig. 2). In a study examining different breeds of cultivated rice, large basal pore size resulted in increased self-pollen deposited on the stigma (Matsui and Kagata 2003). In *O. rufipogon*, anthers hang in a pendent-like fashion, below the flower (Figs 1 and 4). In this case, pollen is predominantly dispensed through the apical pore due to gravity. Alternatively, anthers of *O. nivara* remain upright and pollen falls through the basal pore. The basal pore of *O. rufipogon* is quite small and often does not open, as it likely serves no function (Fig. 2). In these species, the stamen filaments are the same length, and the position of anthers upon anthesis (pendent or upright) is a likely result of pollen mass. Furthermore, in *O. nivara,* the basal pore opens and pollen is released before the flower opens, contributing to prior selfing (Fig. 4). Prior selfing is autonomous selfing that occurs before the opportunity for the receipt of outcross pollen (Lloyd and Schoen 1992). In animal-pollinated systems, this happens due to early spatial and developmental overlap between male and female functions and can occur prior to anthesis (Fishman and Wyatt 1999, Fishman *et al*. 2002). Matsui and Kagata (2003) observed early dehiscence in cultivated rice. Similarly, *O. nivara* flowers that are artificially opened reveal considerable pollen deposited on the stigma (Fig. 1). This phenomenon is not observed in *O. rufipogon* and is likely a necessary step for autogamous selfing in *O. nivara.* Typically, anthesis in wind-pollinated taxa is highly synchronous and abbreviated when compared to animal-pollinated systems (Dowding 1987). Accordingly, pollen of wind-pollinated species is only viable for a short duration (Dafni and Firmage 2000). Pollen viability in *O. nivara* decreased rapidly within 45 minutes (post anther dehiscence), whereas *O. rufipogon* maintained viability (Supplemental Fig. 1). This observation is consistent with results presented by Song *et al*. (2004), in which *O. rufipogon* was approximately 38% viable after 20 minutes, compared to 0% in *Oryza sativa*. Both *O. nivara* and *O. sativa* are highly selfing and are unlikely to experience selection for prolonged pollen viability. Conversely, *O. rufipogon* pollen retains high viability for longer periods allowing for successful fertilization after pollen dispersal. Song *et al*. (2001) identified similar levels of pollen viability for *O. rufipogon*. Grasses possess unique feathery stigmas that are exserted from the flower (Friedman and Harder 2004). Wind tunnel experiments have demonstrated that feathery stigmas have higher pollen collection efficiencies than solid stigmas, as they maintain a high surface area, but produce a thinner boundary layer (Niklas 1985). Stigmas in the focal species differ in their papillae number and pigmentation (Figs 1 and 2). *Oryza rufipogon* has stigmas with a higher density of papillae than *O. nivara*, which may aid in capturing outcross pollen. Additionally, stigmas of *O. rufipogon* are dark purple in coloration, due to the presence of anthocyanins (Fig. 1). Anthocyanins can function to protect plant tissue from environmental stress, including drought and oxidative damage from UV exposure (Stapleton and Walbot 1994, Hughes *et al*. 2010). In order to ensure successful pollen germination, stigmas must maintain a specialized enzymatic surface. Wind-pollinated stigmas are likely prone to desiccation and UV radiation by being exserted outside the flower. Stigma pigmentation in *O. rufipogon* may be an adaptation to promote successful pollen germination and outcrossing. Stigmas in two of the three *O. nivara* accessions lack pigmentation and are white. As a selfer, *O. nivara* would not experience selection to maintain pigmentation, and allocating resources to anthocyanins could incur a cost. Variation in stigma colour is quite common across grass species (Grillo per. obs.) and may potentially be associated with the mating system. Grasses display a range of inflorescence architectures, ranging from diffuse to compact, representing different means for pollen removal and receipt (Friedman and Harder 2005, Friedman and Barrett 2009b). Wind tunnel experiments have demonstrated that compact panicles act to obstruct airflow, thereby creating eddies on the leeward side of the panicle. This allows pollen to settle out of the airstream and become deposited on stigmas. Alternatively, diffuse panicles disturb airflow less, and pollen simply comes in direct contact with windward stigmas (Niklas 1987). By disturbing airflow less, diffuse panicles may be more effective at pollen dispersal (Friedman and Harder 2005). *Oryza rufipogon* accessions have a very open panicle shape which may promote pollen dispersal (Fig. 2). Conversely, *O. nivara* panicles are quite compact. Certainly, the close proximity of simultaneously open flowers within a compact panicle presents an opportunity for geitonogamy. In addition to inflorescence architecture, plant height can influence pollination efficiency. In general, horizontal wind speed increases with height above ground (Burd and Allen 1988). Therefore, taller plants experience higher wind speeds than shorter ones in the same habitat. For this reason, grasses generally protrude their inflorescence above the boundary layer of their foliage (i.e. panicle exsertion) to promote pollen dispersal (Friedman and Harder 2004, 2005). Tiller length is greater in *O. rufipogon* (Grillo *et al*. 2009), although this may not affect plant height as *O. rufipogon* inhabits marshes and tillers essentially float. Panicle exsertion is likely more important in this regard for the focal species. Panicles of *O. nivara* have limited exsertion (Figs 1 and 2) and are essentially nested within the foliage, which is unlikely to provide effective pollen dispersal. The reverse is true for *O. rufipogon*, consistent with an outcrossing mating system.

### Geitonogamy and autogamy

Self-compatible plants with many flowers in anthesis simultaneously present an opportunity for geitonogamous selfing (Dejong *et al*. 1993). This is surely the case in grasses that display highly synchronous bouts of flowering and often have large plant sizes (Friedman and Barrett 2009b). For this reason, it is suspected that avoidance of geitonogamy has driven the common occurrence of dioecy and self-incompatibility in grasses (Friedman and Harder 2005, Friedman and Barrett 2009b). Nonetheless, there are certainly numerous self-compatible hermaphroditic grasses that are prone to this mode of selfing. In order to distinguish between geitonogamy and autogamy, manipulative experiments of floral number are needed (Schoen and Lloyd 1992). Such experiments are rarely carried out, but the few examples reveal high levels of geitonogamous selfing (Harder and Barrett 1995, Eckert 2000, Dorken *et al*. 2002, Karron *et al*. 2004). We have demonstrated through single-floret experiments that in *O. nivara* high levels of selfing are achieved through autogamy. In contrast, self-fertilization in *O. rufipogon* is due to geitonogamy and increases with floret number (Fig. 3). In *O. nivara*, multiple traits act in concert to provide autogamous self-fertilization: namely the timing of anther dehiscence and anther basal pore size. Before the floret even opens, the anthers of *O. nivara* become dehiscent with the opening of the basal pore (Fig. 4). Pollen is released through the basal pore which is quite large in this species. Upon anthesis, the anthers in *O. nivara* remain upright and continue to deposit pollen onto the stigma of the same floret. Thus, autogamy is achieved in *O. nivara* through prior selfing.

### Mating system classification

The development of polymorphic markers and statistical methodologies has allowed for the reliable estimation of outcrossing rates (t) in plants (Ritland and Jain 1981, Ritland 2002, Jantzen *et al*. 2019). It is widely accepted that outcrossing rates below 20% (t ≤ 0.2) and above 80% (t ≥ 0.8) represent predominantly selfing and outcrossing mating systems, respectively. The estimate of the multilocus outcrossing rate in *O. nivara* (t_m_ = 0.04) confirms its status as being predominantly selfing. As an annual with small population sizes (Morishima and Barbier 1990, Vaughan 1994, Zhou *et al*. 2008), selection for reproductive assurance likely drove the evolution of selfing in *O. nivara*. Despite being substantially more outcrossing than *O. nivara*, the mating system estimates from *O. rufipogon* (t_m_ = 0.50 and 0.56) would be classified as mixed-mating (Barbier 1989). It is possible that these estimates may be less than the actual outcrossing rate due to the occurrence of geitonogamy and/or biparental inbreeding. Geitonogamy is thought to be responsible for a significant amount of the variation in outcrossing rates in plants (Barrett 2003). This may be the case in *O. rufipogon*, as we have demonstrated that autogamy is rare in this species and large clonal ramets are common in nature (Xie *et al*. 2001), providing ample opportunity for geitonogamy. Similarly, outcrossing events between close relatives (biparental inbreeding) can result in ‘apparent selfing’, as is observed in self-incompatible species with outcrossing estimates of <1 (Ritland 1984, Goodwillie *et al.* 2005). Populations of *O. rufipogon* can be quite large (Oka and Morishima 1967, Vaughan 1994, Zheng and Ge 2010); however, increasingly their habitats are being fragmented and disturbed, and large populations (>33 ha) have either disappeared or decreased dramatically (Wang *et al*. 2020). For example, Akimoto *et al*. (1999) have monitored one such population in Thailand that became severely fragmented. During a 10-year period, the observed outcrossing rate decreased from 0.538 to 0.236, due in part to an increase in biparental inbreeding. Outcrossing rates should be measured in more populations of each species with genome-wide markers to better estimate the mating system. Regardless, *O. rufipogon* possess attributes consistent with an outcrossing strategy, despite the observed estimates that would be considered mixed-mating.

### Evolution of self-fertilization

Based on the properties of the mating system traits, it is possible to predict the order in which they may have evolved. There are two possible alternatives for the evolution of autogamy: (a) an indirect path where an increase in selection for geitonogamy provides a bridge to autogamy or (b) the direct evolution of autogamy. Selfing likely became beneficial in this system concurrently with a shift to seasonally dry habitats that do not allow plants to persist as perennials. Populations of *O. nivara* are often quite small and potential mates can be scarce. Under such conditions, there would be selection for reproductive assurance through selfing. A similar situation is observed in populations that exhibit increased selfing rates along range margins, where there are few mates available (Barrett 2003, Pannell *et al*. 2015). For the *O. rufipogon*-like ancestor that gave rise to *O. nivara*, selfing would be achieved through geitonogamy. Changes in inflorescence architecture could influence the rates of geitonogamy, and variation for this trait is common in natural populations. Selection for compact panicles would thereby provide close proximity of florets and aerodynamic properties favourable for high geitonogamous selfing. Once appreciable levels of self-fertilization were achieved through geitonogamy, there would be selection for reduced investment in allocation to male function. Large anthers are likely to be energetically costly and excess pollen production (for outcrossing) would be unnecessary under these conditions. As anthers began to shorten, they would weigh less and maintain a more upright position. At this point, gravity would no longer allow pollen to exit through the apical pore; rather the basal pore would be more important in this regard. Selection for large basal pores would allow pollen to fall directly onto the stigma of the same floret, thus providing autogamous selfing. After this point, alterations in the timing of anther dehiscence would result in prior selfing. In this way, geitonogamy would serve as a bridge to autogamous selfing. Alternatively, autogamy may have evolved directly in an *O. rufipogon*-like ancestor. In this scenario, selfing would have to occur prior to anthesis, as the large basal pores of *O. rufipogon* would fall below the flower upon opening. Therefore, anther dehiscence would be the first trait to evolve. Early anther dehiscence would be unlikely to provide reliable autogamous selfing alone and would have to be accompanied closely by an increase in basal pore size to deposit pollen on the stigma. Once autogamous selfing became predominant, there would be selection for reduced anther size. In either case, traits that promote outcrossing are expected to evolve after autogamy has been established. These traits include pollen viability and stigma characteristics (length, papillae density, pigmentation).

## Conclusion

Wind-pollinated plants represent some of the most ecologically and economically important plants on the planet. To our knowledge, this is the first study of comparative floral biology between closely related wind-pollinated species that are distinct in their mating system. Thus, this system can serve as a model for understanding the mechanisms of wind pollination and mating system evolution, particularly for Poaceae. From our analysis, the large basal pore of the anther and early dehiscence function provide reliable autogamous selfing. Perhaps these traits are analogous to stigma-anther separation, which provides a reliable means of autogamous selfing in numerous animal-pollinated groups. Future experiments should examine the nature of anther dehiscence in other selfing and outcrossing species to determine how universal these mechanisms are across wind-pollinated systems. This experimental framework may be particularly useful when studying self-fertilized cereal crops and their wild outcrossing congeners.

## Supplementary Material

plae073_suppl_Supplementary_Figure

plae073_suppl_Supplementary_Table_S1

plae073_suppl_Supplementary_Table_S2

## Data Availability

All phenotypic trait data is provided in Supplemental Table 1 and code for statistical analyses is available on the Grillo lab Github (https://github.com/grillolab/Oryza-mating-system-study).
