# Supplementary material for: Floral traits underlying mating system differentiation in the wind-pollinated sister species Oryza rufipogon and Oryza nivara
Source: AoB Plants. 2024 Dec 31;17(1):plae073. doi: 10.1093/aobpla/plae073 (PMC11752648; doi:10.1093/aobpla/plae073)
Supplement: plae073_suppl_Supplementary_Figure [file plae073_suppl_supplementary_figure.pdf]

Supplemental Figure 1

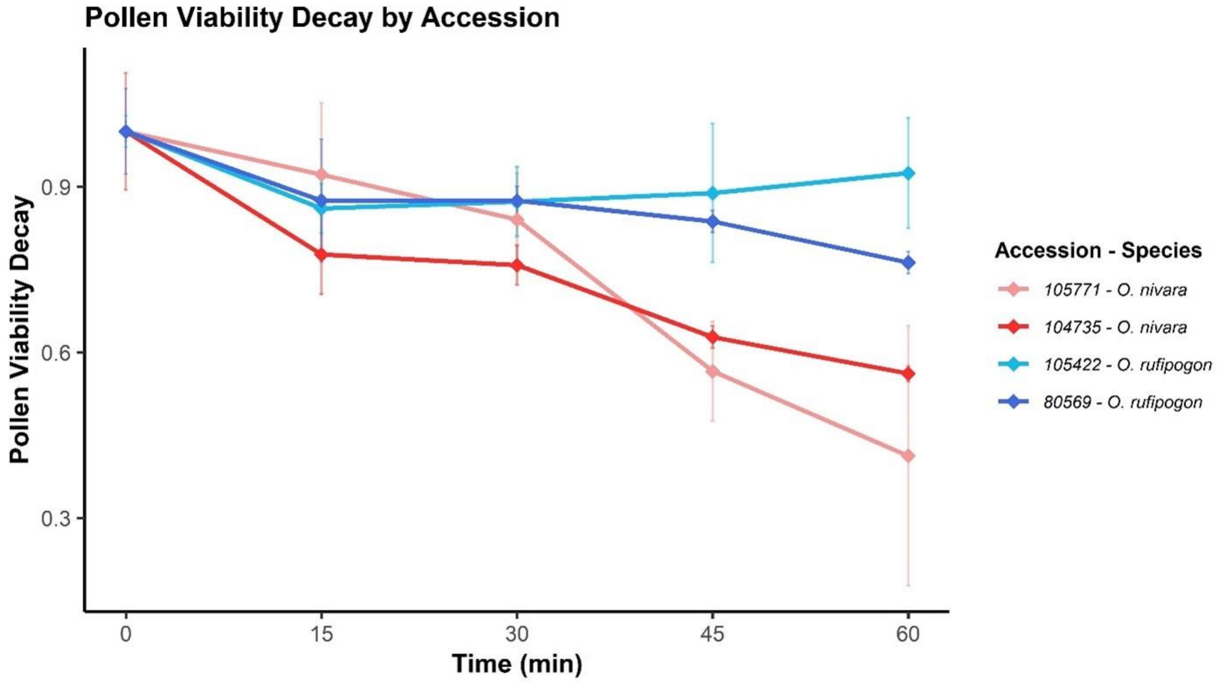


Supplemental Figure 1. Pollen viability time course for *O. nivara* and *O. rufipogon* showing the percent decrease in pollen viability post anther dehiscence.
