# Supplementary material for: Floral traits underlying mating system differentiation in the wind-pollinated sister species Oryza rufipogon and Oryza nivara
Source: AoB Plants. 2024 Dec 31;17(1):plae073. doi: 10.1093/aobpla/plae073 (PMC11752648; doi:10.1093/aobpla/plae073)
Supplement: plae073_suppl_Supplementary_Table_S2 [file plae073_suppl_supplementary_table_s2.docx]

Supplemental Table 2: Summary of the multivariate analyses of variance results comparing floral traits between *Oryza rufipogon* and *O. nivara*.

| **Dependent variables** | **MANOVA (p-value)** | | **ANOVA results (p-value)** | |
| --- | --- | --- | --- | --- |
|  | **Among accessions** | **Across species** | **Among accessions** | **Across species** |
| Anther length (mm) | <0.0001 | <0.0001 | <0.0001 | <0.0001 |
| Basal pore diameter (mm) |  |  | <0.0001 | <0.0001 |
| Panicle exsertion (cm) | <0.0001 | <0.0001 | 0.004 | <0.0001 |
| Panicle angle |  |  | <0.0001 | <0.0001 |
| Papillae density (#fil/mm) | <0.0001 | 0.0007 | 0.0186 | 0.0006 |
| Stigma length (mm) |  |  | 0.0063 | 0.0001 |
